# Advancing Patient Advocacy in Mycology: Cultivating Collaboration in Education, Research, and Policy

**DOI:** 10.1007/s11046-024-00906-6

**Published:** 2025-01-08

**Authors:** Rob Purdie, Rasha Kuran, Ana Alastruey-Izquierdo, Hatim Sati, Juan Luis Rodriguez-Tudela, Martin Hoenigl, John Perfect, Rita Oladele, Thomas J. Walsh, Tom Chiller

**Affiliations:** 1 MyCARE Foundation, Colorado Springs, CO USA; 2 Taskforce of AMR Survivors, World Health Organization, Geneva, Switzerland; 3 Valley Fever Institute, Kern Medical, Bakersfield, CA USA; 4 Mycology Reference Laboratory, National Centre for Microbiology, Instituto de Salud Carlos III, Madrid, Spain; 5 Department of Global Coordination and Partnership on Antimicrobial Resistance, World Health Organization, Geneva, Switzerland; 6 Global Action for Fungal Infections, Rue Le Corbusier 12, 1208 Geneva, Switzerland; 7 Division of Infectious Diseases, ECMM Excellence Center of Medical Mycology, Department of Internal Medicine, Medical University of Graz, Graz, Austria; 8 Infectious Diseases, Duke University, Durham, NC USA; 9 College of Medicine, University of Lagos, Lagos, Nigeria; 10 Center for Innovative Therapeutics and Diagnostics, Richmond, VA USA; 11 Departments of Medicine and Microbiology and Immunology, School of Medicine, University of Maryland, Baltimore, MD USA; 12 Mycotic Diseases Branch, Centers for Disease Control and Prevention, Atlanta, GA USA

**Keywords:** Fungal disease, Mycology, Fungus, Patient advocacy, Lived experience, AMR, Antimicrobial resistance

## Abstract

In the healthcare landscape, diseases such as cancer and HIV/AIDS have benefited from the patient's perspective. For fungal diseases, the patient voice remains absent in critical areas such as policy formulation, funding decisions, and research priorities. Patients affected by fungal disease, along with their caregivers and advocacy groups, possess invaluable insights into the challenges and unmet needs they face. By elevating their voices and experiences, policymakers, funding agencies, and researchers can gain a more comprehensive understanding of the multifaceted nature of fungal disease and the urgency of addressing them. This paper addresses the pressing need for a coordinated effort to elevate the patient voice in advocating for improved policies, increased funding, and enhanced research initiatives regarding fungal disease.

## Introduction

In the healthcare landscape, diseases like cancer and HIV have benefited from the pivotal role of patient advocacy in shaping policies, funding allocations, and research endeavors. However, for other diseases, patient advocacy remains absent. This discrepancy is especially pronounced in the area of fungal diseases, which despite their prevalence and profound impact on public health, have often been relegated to the sidelines in discussions surrounding infectious disease and anti-infective priorities.

Fungal diseases encompass a spectrum of conditions, ranging from superficial skin ailments to life-threatening systemic illnesses, imposing a significant burden on individuals, healthcare systems, and societies at large. Invasive fungal diseases (IFDs) encompass a range of acute, life-threatening infections as well as chronic, debilitating conditions that primarily affect severely immunocompromised individuals, such as cancer patients and organ transplant recipients. For instance, mold infections, including Aspergillus and Fusarium, can rapidly escalate in patients with weakened immune systems, often leading to high mortality rates if not promptly diagnosed. The emergence of *Candida auris* as a multidrug-resistant pathogen has fueled outbreaks, highlighting the rising threat of hospital-associated fungal infections. Dimorphic fungi, such as *Histoplasma, Blastomyces*, and *Coccidioides*, pose additional risks; often infecting healthy individuals, they can cause severe respiratory and systemic diseases, especially if the immune system is compromised. Mucormycosis, infamously linked to COVID-19, has shown how these pathogens exploit weakened host defenses. Together, these examples underscore the need for greater awareness and research to improve patient outcomes.

Yet, compared to their bacterial counterparts, fungal diseases have historically received inadequate attention in policy agendas, funding streams, and research initiatives [1]. This disparity is evident in the limited resources allocated to the prevention, diagnosis, and treatment of fungal diseases, as well as the scarcity of dedicated research efforts aimed at understanding the complexities of fungal pathogens and developing effective interventions.

There is an urgent need for a concerted and coordinated effort to include the patient voice in advocating for enhanced recognition, resources, and support for fungal disease-related issues. Those infected by fungal diseases, along with their caregivers possess invaluable insights into the challenges and unmet needs within the healthcare system [2, 3]. By including their voices and experiences, policymakers, funders, and researchers can gain a more comprehensive understanding of the multifaceted nature of fungal diseases and the urgent need to address them.

This paper discusses the pressing need for a coordinated effort to advocate for improved policies, increased funding, and enhanced research initiatives to address the increasing impact of fungal diseases. By fostering a culture of inclusivity and actively engaging those with firsthand experience, healthcare stakeholders can ensure that the unique challenges posed by fungal diseases can no longer be overlooked. Through strategic partnerships we can collectively work towards advancing the prevention, diagnosis, and treatment of fungal diseases, while improving outcomes and quality of life for affected individuals.

## The Patient Voice: Advocacy in Policy, Funding, and Research

At the heart of the issue lies the lack of a cohesive and unified strategy among stakeholders involved in fungal disease advocacy [4, 5]. While various initiatives exist, the absence of a unified strategy undermines their effectiveness. Without a concerted effort to engage policymakers; progress stalls allowing crucial opportunities for policy reform and funding allocation to pass. Furthermore, insufficient awareness among those at greatest risk, the public, and policymakers compounds the problem by perpetuating the cycle of neglect [6]. The core of the ignorance surrounding fungal diseases is the absence of advocacy efforts like those in the cancer and HIV communities. Despite the desire for advocacy and awareness initiatives, a lack of collaboration among stakeholders hampers the effectiveness of individual efforts and diminishes the collective strength of the advocacy community.

Misconceptions and lack of understanding about the severity and prevalence of fungal diseases contribute to their marginalization in healthcare policy and funding priorities. Advocacy efforts must prioritize raising awareness and disseminating accurate information about fungal infections to dispel myths and misconceptions. By educating those diagnosed with fungal diseases, healthcare providers, and policymakers about the burden of disease and the need for increased investment in research and treatment, advocates can mobilize support for policy changes and funding initiatives.

Another critical barrier to progress in mycology is the lack of engagement with policymakers. Without a concerted effort to communicate the urgency of addressing the increasing impact of fungal diseases, opportunities for policy reform and funding allocations remain unrealized. Effective advocacy requires initiative-taking engagement with policymakers at local, national, and international levels to ensure that fungal diseases are appropriately prioritized in the healthcare agenda. By articulating the impact of fungal diseases on individuals, communities, and healthcare systems, advocates can compel policymakers to act and allocate resources to address this neglected public health challenge.

The fragmented landscape of fungal disease funding, research and advocacy initiatives underscores the pressing need for a common plan that unites stakeholders under a shared vision. By fostering collaboration and consensus among diverse stakeholders, including healthcare providers, researchers, policymakers, and advocacy groups, we can harness collective expertise and resources to drive transformative change.

## Addressing Messaging Gaps and Awareness Deficits

In the realm of public health communication, effective messaging plays a pivotal role in shaping perceptions, driving awareness, and mobilizing action. However, when it comes to fungal diseases and antimicrobial resistance (AMR), there exists a notable deficiency in the consistency and comprehensiveness of messaging strategies. This deficiency stems from the use of terminology that inadequately captures the breadth and complexity of the issue, thereby hindering efforts to convey its urgency and significance to both the public and policymakers.

One critical challenge lies in the terminology used to describe antimicrobial resistance. While terms like "antibiotic resistance," "superbugs," and "AMR" are commonly employed, they evoke associations with bacterial pathogens, overlooking the equally pressing threat posed by fungal and other non-bacterial infections. This limited scope fails to convey the full extent of the problem and may inadvertently contribute to underestimation of the risks associated with fungal infections.

Additionally, publications and papers often fall short in effectively conveying the gravity of the situation to both the public and policymakers. The failure to articulate the comprehensive threat posed by antimicrobial resistance, encompassing fungal diseases alongside bacterial, hampers efforts to garner support for initiatives aimed at combating this global health crisis [7]. To address the messaging gaps and awareness deficits, a concerted effort is needed to develop and disseminate clear, consistent, and inclusive messaging. This entails adopting terminology that accurately reflects the diverse array of pathogens involved in antimicrobial resistance, including fungi, and emphasizes the interconnected nature of the issue.

## Legislative and Funding Limitations

The challenge of addressing fungal diseases and antimicrobial resistance (AMR) is further exacerbated by the prevailing legislative and funding frameworks that focus on bacterial infections. Initiatives such as CARB-X (Combating Antibiotic-Resistant Bacteria Biopharmaceutical Accelerator) and ARPA-H (Advanced Research Projects Agency for Health) Dart are commendable efforts aimed at combating antibiotic resistance, yet they exclude fungal pathogens. While these initiatives have yielded significant advancements in bacterial infection control, they inherently overlook the multifaceted nature of AMR, which encompasses fungal and other non-bacterial infections.

The disproportionate emphasis on bacterial pathogens in legislative and funding priorities reflects a systemic bias that undermines efforts to address the broader spectrum of infectious diseases. This narrow focus perpetuates a cycle of neglect surrounding fungal diseases, relegating them to the sidelines of policy agendas and funding allocations. Consequently, resources earmarked for research, development, and implementation of interventions disproportionately favor bacterial-centric approaches, leaving critical gaps in addressing the evolving threat posed by fungal pathogens.

The absence of fungal advocates in discussions surrounding legislative and funding priorities further compounds the issue. Patients and caregivers, as key stakeholders directly impacted by fungal infections and AMR, are notably excluded from decision-making processes, resulting in policies and funding priorities that inadequately reflect their needs and perspectives. This oversight not only undermines the legitimacy of these initiatives but also perpetuates disparities in access to care and resources for affected individuals.

To address these limitations, there is a critical need for legislative and funding frameworks that adopt a comprehensive and inclusive approach to AMR. This entails recognizing fungal diseases as a significant component of the broader antimicrobial resistance landscape and allocating resources accordingly. Furthermore, meaningful engagement of patient advocacy groups in policy formulation and funding decisions is essential to ensure that interventions are tailored to meet the diverse needs of affected individuals.

By broadening the scope of legislative and funding priorities to encompass fungal diseases and incorporating patient advocates into decision-making processes, stakeholders can foster more equitable and effective strategies for combating AMR. This requires a change in basic assumptions towards recognizing the interconnectedness of infectious diseases and prioritizing One Health approaches that address the full spectrum of pathogens contributing to antimicrobial resistance.

## The Role of Patient Advocacy and Collaboration

To bridge these gaps, there is an urgent need for a dedicated non-governmental organization (NGO) to serve as the common thread among stakeholders and the public. Such an organization would facilitate collaboration, amplify patient voices, and advocate for policies and funding that reflect the broader spectrum of infectious diseases, including fungal infections. Central to this effort is the establishment of a comprehensive strategy to engage policymakers, increase awareness, and drive meaningful change.

## The MyCARE (MyCology, Advocacy, Research, & Education) Mission: Empowering Patients in Mycology Advocacy

A promising initiative in this regard is the MyCARE Foundation, which seeks to bring mycology advocates to the table to improve outcomes through advocacy, policy, research, and education. By centering patient perspectives and experiences, MyCARE aims to fill existing gaps in fungal advocacy and AMR messaging. Through strategic collaborations with healthcare professionals, professional societies, industry stakeholders, and other advocacy groups, MyCARE endeavors to amplify patient voices and effect positive change.

## Conclusion

In conclusion, patient advocacy organizations are a powerful yet underutilized force in shaping policy, funding, and research initiatives related to fungal diseases and antimicrobial resistance. There are many lessons that can be learned from the advocacy efforts of the AIDS community in the 1980s. While employing tactics that should not be emulated, organizations like Act Up brought the AIDS epidemic to the attention of the entire nation and changed the way that the FDA viewed clinical trials for new medications for HIV/AIDS. Legislation named in honor of Ryan White continues to fund research and ensure that those diagnosed with HIV have access to care and therapeutics.

National initiatives like the Cancer Moonshot under the Biden Administration increased funding for research as well as navigation and supportive services to improve outcomes for those battling cancer. The numerous charities and nonprofits of the cancer community have raised billions to support cancer research and aid patients at a level that has not occurred with any other group of diseases.

Organ donor registries have increased the number of organ donors and increased opportunities for positive outcomes for those in need of organ transplants.

By addressing the lack of collaboration, inconsistent messaging, and awareness deficits, we can begin to effect meaningful change. Through initiatives like MyCARE and concerted efforts to engage policymakers and stakeholders, we can establish advocacy to increase resources for the mycology community and drive progress towards improved outcomes for all. It is time to recognize the pivotal role of those who have firsthand experience with fungal diseases in shaping the future of advocacy and AMR mitigation efforts.
